# Supplementary material for: Formal Statistical Replication Analysis in Lung Cancer Genome-Wide Association Studies
Source: medRxiv. 2025 Oct 3:2025.10.02.25337130. Preprint. [Version 1] doi: 10.1101/2025.10.02.25337130 (PMC12622082; doi:10.1101/2025.10.02.25337130)
Supplement: Supplement 1 [file media-1.pdf]

Supplementary Materials

Appendix A: Simulation Results When Using Parameters Similar to ILCCO Real Data

Appendix B: Two-Way Replication Analysis Results for ILCCO and UKB

Appendix C: Replication Results for Top Sentinel SNPs from ILCCO GWAS

Appendix A: Simulation Results When Using Parameters Similar to ILCCO Real Data

	number of significant			Power			FDR		
**% of causal variants**	0.02	0.2	1	0.02	0.2	1	0.02	0.2	1
Replication Analysis	461.20	9339.46	58676.68	0.315	0.625	0.779	0.079	0.098	0.105
Meta Analysis	318.19	2893.57	14299.91	0.211	0.210	0.211	0.107	0.019	0.006
$P < 10^{-8}$	0.11	1.06	5.43	8.16×10^{-5}	7.86×10^{-5}	8.05×10^{-5}	-	-	-
$P < 10^{-6}$	2.40	25.83	126.12	1.74×10^{-3}	1.90×10^{-3}	1.87×10^{-3}	-	0.006	0.001
$P < 10^{-5}$	10.76	105.40	521.36	7.44×10^{-3}	7.77×10^{-3}	7.70×10^{-3}	0.070	0.013	0.004

Supplementary Table 1: Simulation results for two-way replication study under parameters of ILCCO real data. We applied the Expectation-Maximization Algorithm to ILCCO data to estimate the means of summary statistics under the alternative. We also estimated the percentages of causal variants to be approximately 0.2%. We then used the estimated means along with three different causal proportions (0.02%, 0.2%, and 1%) to perform simulations using model-based replication analysis, meta-analysis, and the threshold method. As expected, the model-based replication approach outperforms the other methods in both FDR and power.

Appendix B: Two-Way Replication Analysis Results for ILCCO and UKB

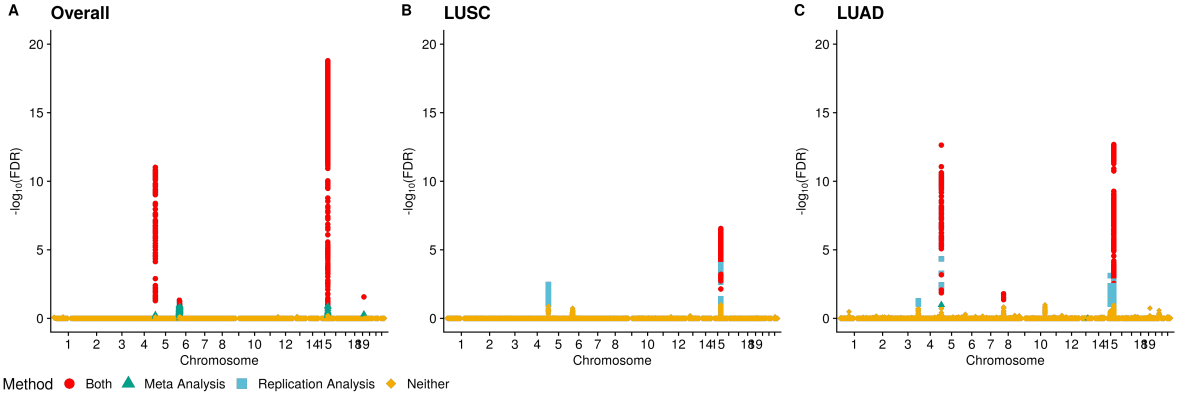

Supplementary Figure 1: *Two-way Replication Analysis Results for ILCCO and UKB.* The y-axis is the $-log_{10}(FDR)$ for SNPs from three different GWAS summary statistics. SNPs prioritized by different methods are highlighted in different colors to illustrate overlap and method-specific signals. Fewer SNPs are replicated in the ILCCO and UKB analysis than the ILCCO and MVP analysis since there is less signal in UKB than in MVP.

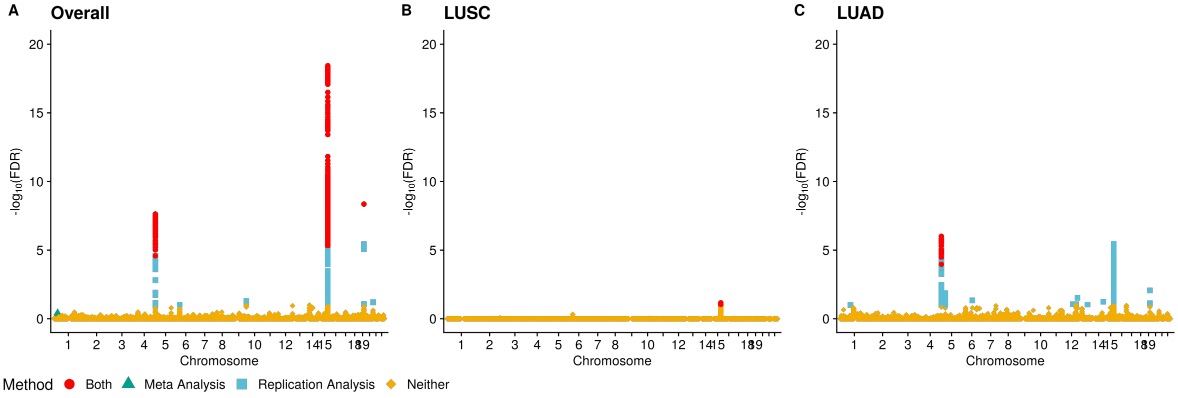

Supplementary Figure 2: *Two-way Replication Analysis Results for UKB and MVP.* The y-axis is the $-log_{10}(FDR)$ for SNPs from three different GWAS summary statistics. SNPs prioritized by different methods are highlighted in different colors to illustrate overlap and method-specific signals. Fewer SNPs are replicated in the MVP and UKB analysis than the ILCCO and MVP analysis since there is less signal in UKB than in ILCCO.

Appendix C: Replication Results for Top Sentinel SNPs from ILCCO GWAS

**McKay's SNPs (2017)**					**Model-based Replication Analysis**		
**RS**	**Gene**	**Cancer**	**Chr**	**BP**	**Overall**	**LUSC**	**LUAD**
rs71658797	FUBP1	Overall	1	77967507	No	No	No
rs6920364	RNASET2	Overall	6	167376466	No	No	No
rs11780471	CHRNA2	Overall	8	27344719	No	No	No
rs11571833	BRCA2	Overall	13	32972626	No	No	No
rs66759488	SEMA6D	Overall	15	47577451	No	No	No
rs55781567	CHRNA5	Overall	15	78857986	**Yes**	**Yes**	**Yes**
rs56113850	CYP2A6	Overall	19	41353107	**Yes**	No	**Yes**
rs13080835	TP63	LUAD	3	189357199	No	No	**Yes**
rs7705526	TERT	LUAD	5	1285974	**Yes**	No	**Yes**
rs4236709	NRG1	LUAD	8	32410110	No	No	No
rs885518	CDNK2A	LUAD	9	21830157	No	No	No
rs11591710	OBFC1	LUAD	10	105687632	No	No	**Yes**
rs1056562	AMICA1	LUAD	11	118125625	No	No	No
rs77468143	SECISBP2L	LUAD	15	49376624	No	No	**Yes**
rs41309931	RTEL1	LUAD	20	62326579	No	No	No
rs116822326	MHC	LUSC	6	31434111	No	No	No
rs7953330	RAD52	LUSC	12	998819	No	No	No
rs17879961	CHEK2	LUSC	22	29121087	No	No	No

Supplementary Table 2: Top sentinel SNPs identified in ILCCO original GWAS (McKay et. al., 2017) along with replication status when using three-way model-based replication analysis with UK Biobank and MVP data. Some top SNPs have very small p-values in all cohorts. However, many less significant sentinel SNPs do not show evidence of association in all cohorts. In other words, while the most significant SNPs in a GWAS are often replicated, less significant variants are more likely to be false positives, which is an expected result.
